# Supplementary figures and images for: Back from the dead; the curious tale of the predatory cyanobacterium Vampirovibrio chlorellavorus
Source: PeerJ. 2015 May 21;3:e968. doi: 10.7717/peerj.968 (PMC4451040; doi:10.7717/peerj.968)

Depth of coverage

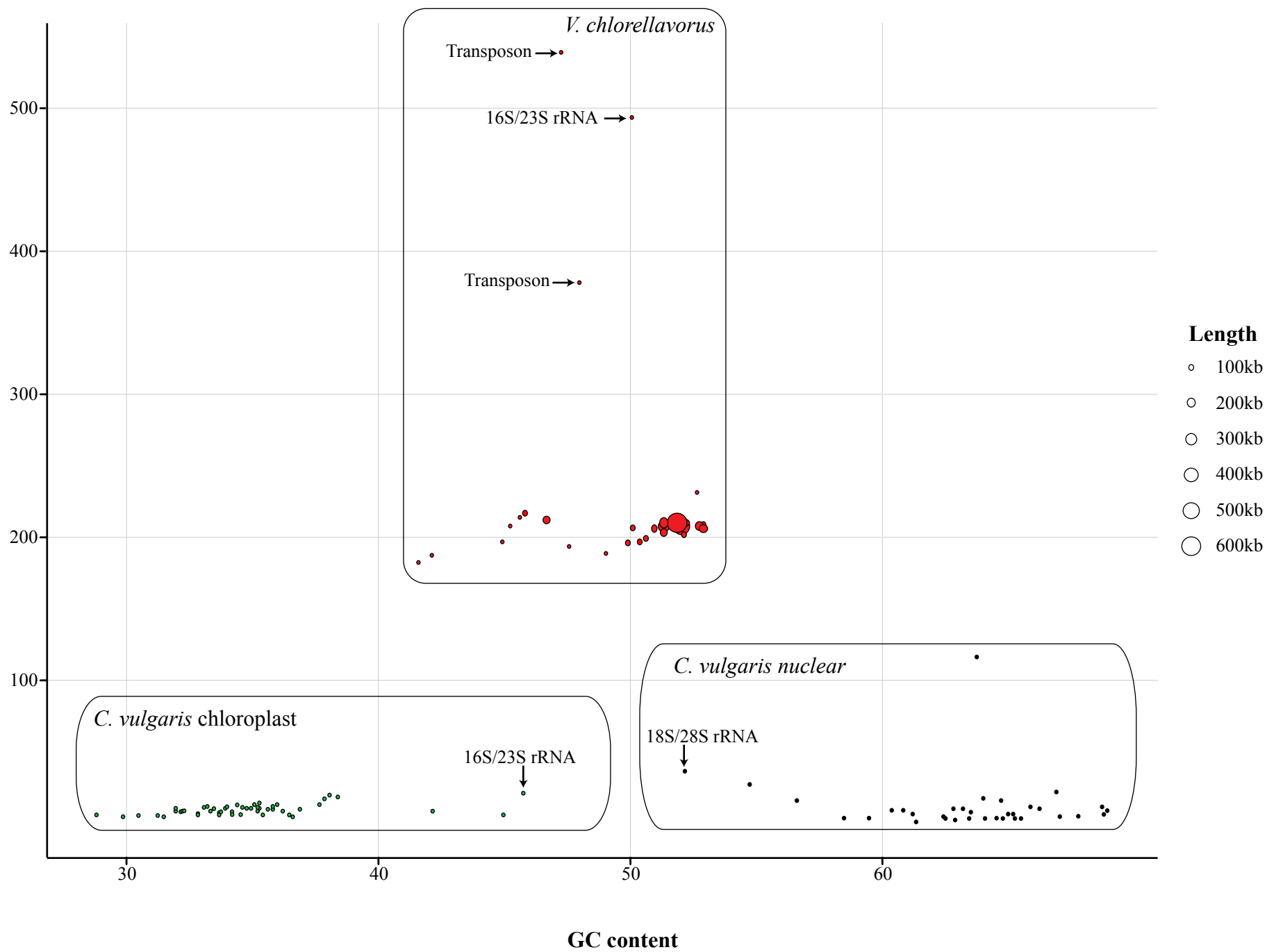

Supplement: Figure S1 — Contigs assigned to V. chlorellavorus are represented by red circles. C. vulgaris chloroplast contigs are represented by green circles and C. vulgaris contigs are represented by black circles. The size of the circle corresponds to the length of the contig. [file peerj-03-968-s001.pdf]

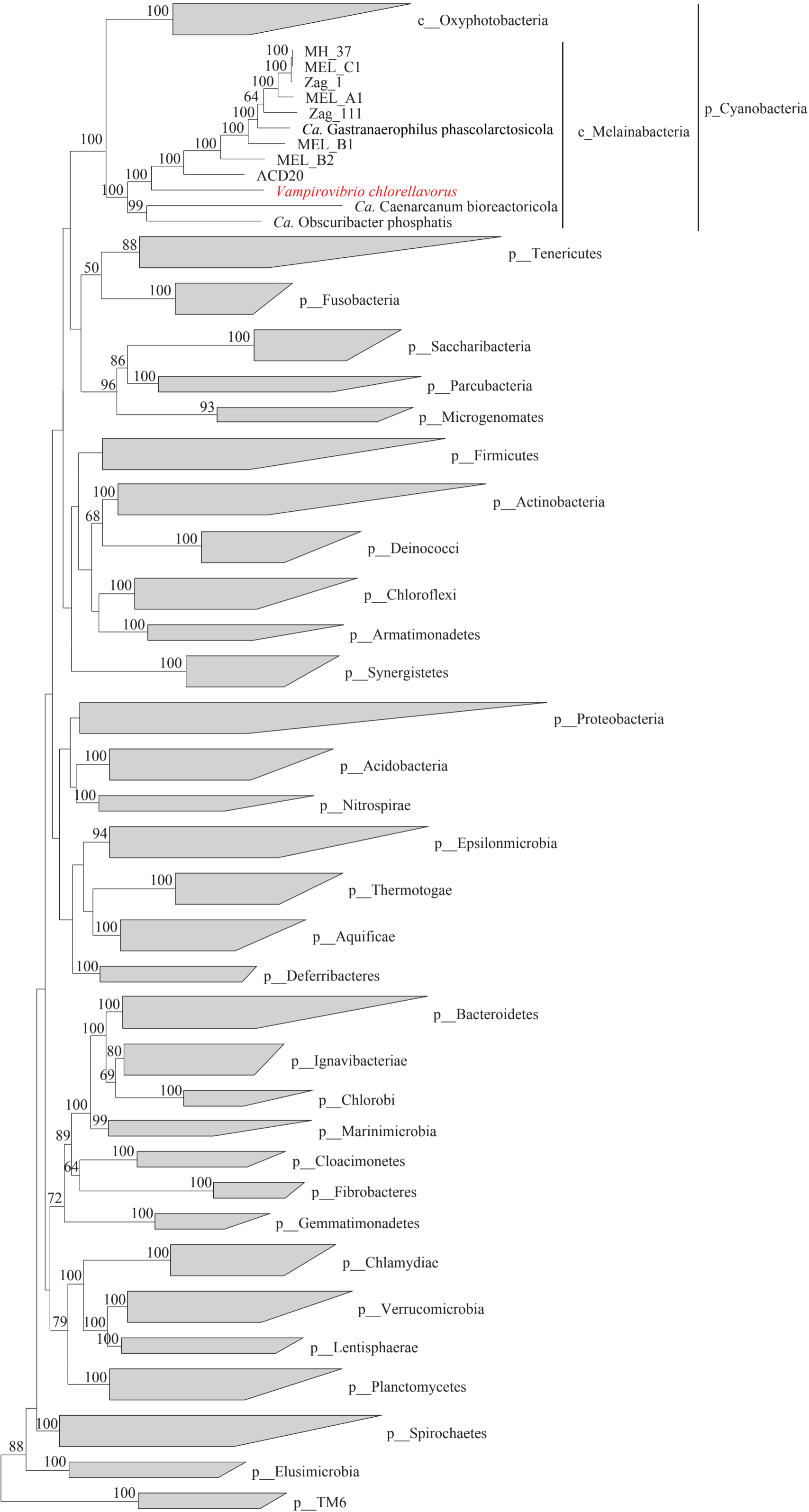

Supplement: Figure S2 — The phylogenetic tree was inferred from the concatenation of 109 conserved marker genes (Table S1) and consists of 7,732 bacterial and 169 arachael genomes from the IMG database (Markowitz et al., 2014). [file peerj-03-968-s002.pdf]

## Contig 21

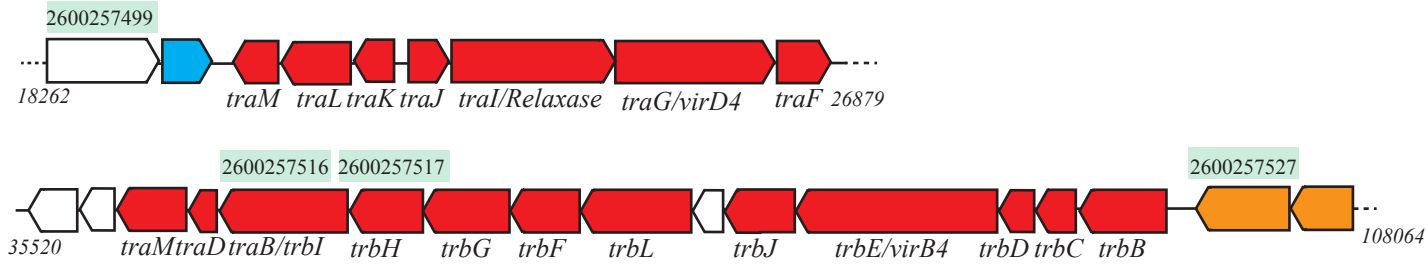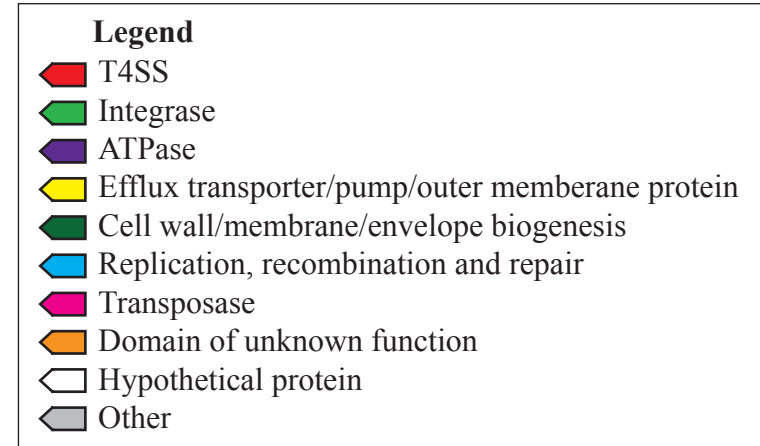

## Plasmid 1

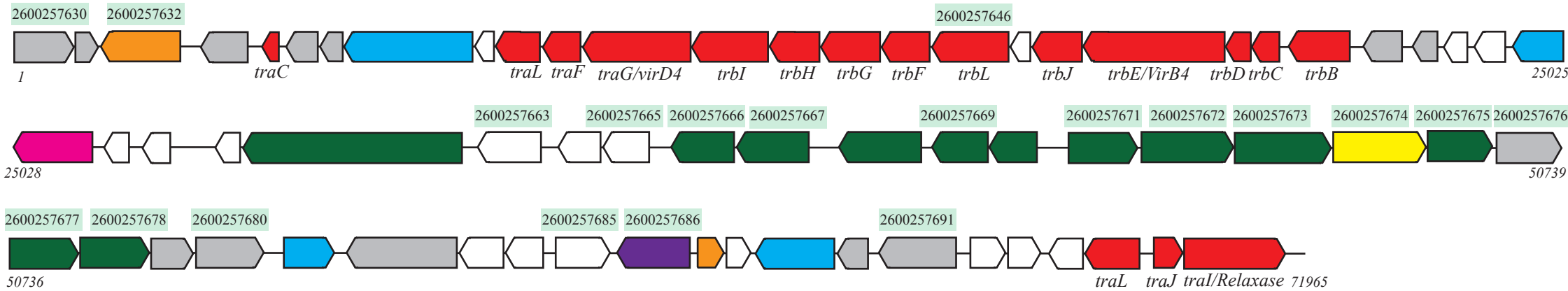

## Plasmid 2

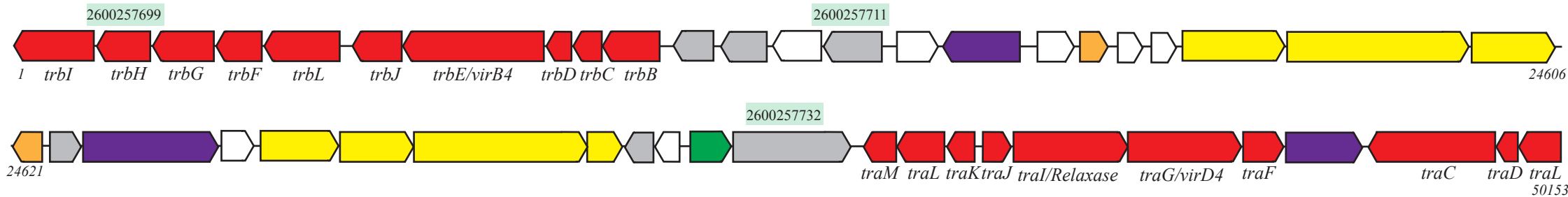

Supplement: Figure S4 — The schematic diagram shows the presence of T4SS genes identified by IMG/ER on both plasmids and one contig. The arrows represent annotated genes and their direction. The numbers above the genes are the IMG/ER accession numbers and these have been identified as alien genes by PHX analysis (Table S2). T4SS genes have the predicted gene names italicised below. [file peerj-03-968-s004.pdf]

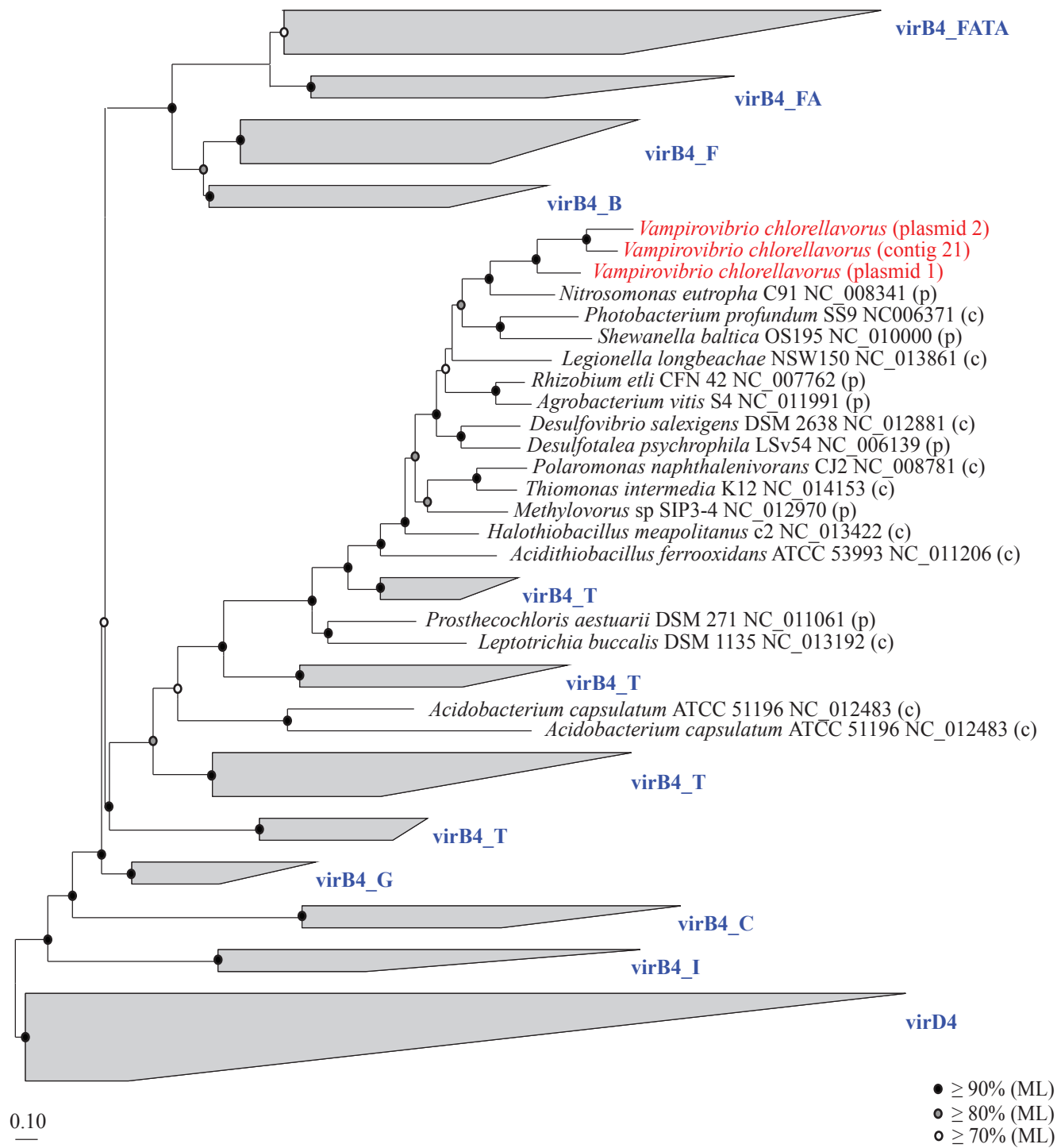

*virB4\_T/trbE*

Supplement: Figure S5 — Aligned sequences and naming conventions were obtained from Christie (2004). virB4_T is based on the T-DNA conjugation system of Agrobacterium tumefaciens plasmid Ti, virB4_F is based on the plasmid F, virB4_I is based on the Incl plasmid R64 and virB4_G is based on ICEHIN1056. The other T4SS have homologues to VirB4 and include the Cyanobacteria (virB4_C), Bacteroides (virB4_B), Firmicutes (virB4_FA and virB4_FATA), Actinobacteria (virB4_FA and virB4_FATA), Tenericutes (virB4_FATA) and Archaea (virB4_FATA) (Christie, 2004). The V. chlorellavorus genome contains T4SS that belong to the virB4_T. virD4 is used as the outgroup. Black circles represent nodes with ≥90% bootstrap support, grey circles represent nodes with ≥ 80% bootstrap support and white circles represent nodes with ≥70% bootstrap support. (p) corresponds to virB4 genes found on plasmids and (c) corresponds to virB4 genes found on the chromosome. [file peerj-03-968-s005.pdf]

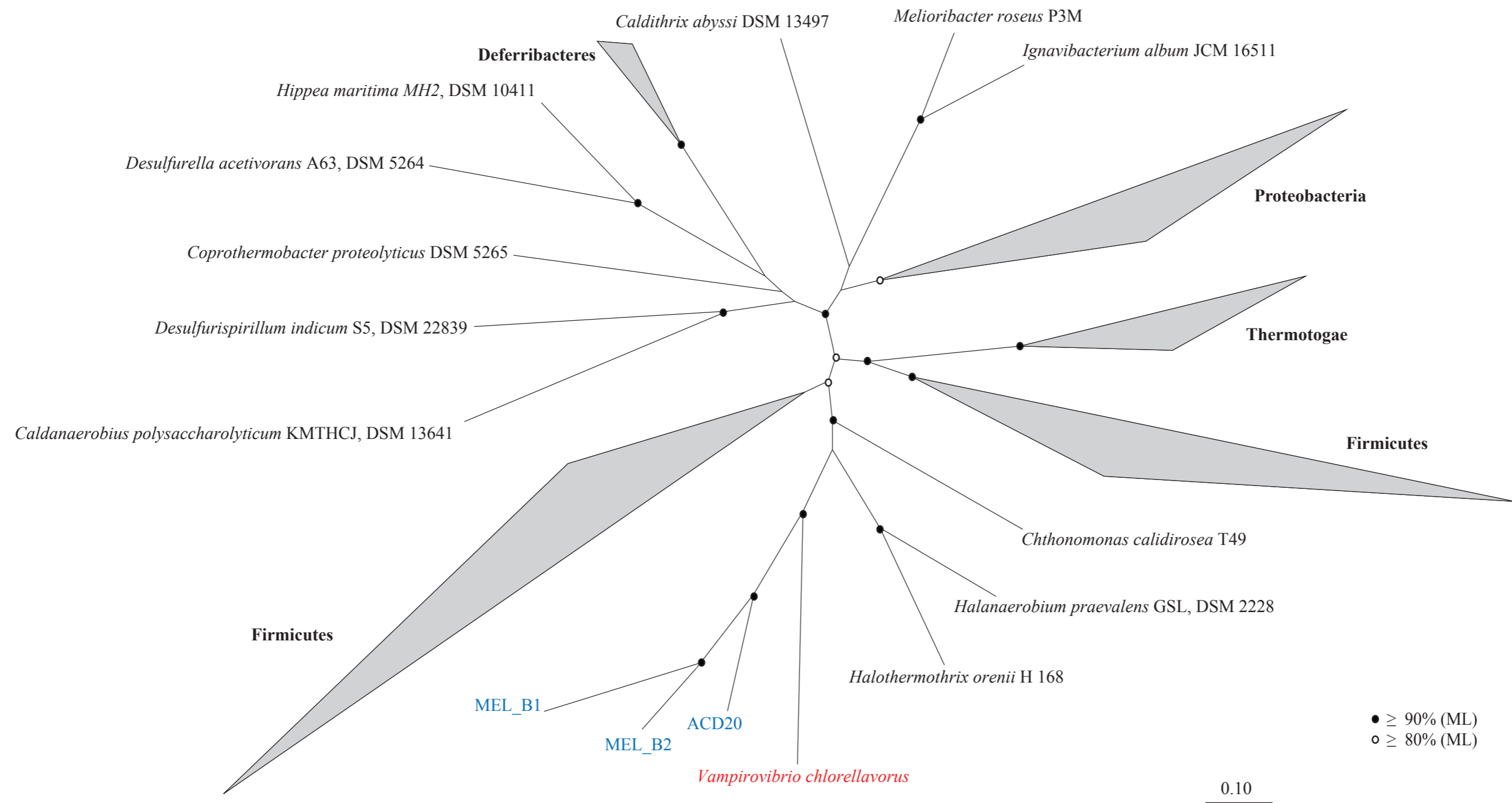

Supplement: Figure S6 — The phylogenetic tree is constructed from 2,256 finished genomes from the IMG database (Markowitz et al., 2009). The tree is unrooted and only the Melainabacteria and its closest neighbours are shown. V. chlorellavorus is in red and the other three Melainabacteria representatives are in blue. Phyla are in bold. Black circles in the tree represents nodes with ≥90% bootstrap support and white circles represents nodes with ≥80% bootstrap support. [file peerj-03-968-s006.pdf]
