# Supplementary material for: Back from the dead; the curious tale of the predatory cyanobacterium Vampirovibrio chlorellavorus
Source: PeerJ. 2015 May 21;3:e968. doi: 10.7717/peerj.968 (PMC4451040; doi:10.7717/peerj.968)
Supplement: Table S1 — A set of 178 single copy genes present exactly once in >90% of the trusted genomes (found in >90% of the genomes) from the Integrated Microbial Genomes (IMG; Markowitz et al., 2014) database was identified. From the 178 initial genes, 69 were removed from consideration as they exhibited divergent phylogenetic histories in >1% of the trusted genomes. The remaining 109 genes were used to construct a concatenated gene tree (Fig. S2). [file peerj-03-968-s008.docx]

| **Gene ID** | **Description** | **Length (aa)** |
| --- | --- | --- |
| **Included markers** | | |
| TIGR03723 | T6A_YgjD: tRNA threonylcarbamoyl adenosine modification protein YgjD | 314 |
| TIGR03953 | rplD_bact: 50S ribosomal protein L4 | 188 |
| TIGR00755 | ksgA: dimethyladenosine transferase | 256 |
| TIGR00138 | gidB: 16S rRNA (guanine(527)-N(7))-methyltransferase GidB | 183 |
| TIGR01953 | NusA: transcription termination factor NusA | 340 |
| PF00410 | Ribosomal protein S8 | 129 |
| PF00380 | Ribosomal protein S9/S16 | 121 |
| TIGR03625 | L3_bact: 50S ribosomal protein L3 | 202 |
| TIGR01049 | rpsJ_bact: ribosomal protein S10 | 99 |
| TIGR01044 | rplV_bact: ribosomal protein L22 | 103 |
| TIGR00964 | secE_bact: preprotein translocase, SecE subunit | 57 |
| TIGR00613 | reco: DNA repair protein RecO | 239 |
| TIGR00002 | S16: ribosomal protein S16 | 78 |
| TIGR00001 | rpmI_bact: ribosomal protein L35 | 63 |
| PF13507 | CobB/CobQ-like glutamine amidotransferase domain | 259 |
| TIGR03591 | polynuc_phos: polyribonucleotide nucleotidyltransferase | 689 |
| TIGR03594 | GTPase_EngA: ribosome-associated GTPase EngA | 432 |
| TIGR00088 | trmD: tRNA (guanine(37)-N(1))-methyltransferase | 233 |
| TIGR00086 | smpB: SsrA-binding protein | 144 |
| TIGR00084 | ruvA: Holliday junction DNA helicase RuvA | 192 |
| TIGR00082 | rbfA: ribosome-binding factor A | 115 |
| TIGR00855 | L12: ribosomal protein L7/L12 | 125 |
| TIGR01032 | rplT_bact: ribosomal protein L20 | 114 |
| TIGR00019 | prfA: peptide chain release factor 1 | 361 |
| TIGR00396 | leuS_bact: leucine--tRNA ligase | 843 |
| TIGR00012 | L29: ribosomal protein L29 | 56 |
| TIGR00017 | cmk: cytidylate kinase | 217 |
| TIGR00150 | T6A_YjeE: tRNA threonylcarbamoyl adenosine modification protein YjeE | 135 |
| TIGR00152 | TIGR00152: dephospho-CoA kinase | 188 |
| TIGR00158 | L9: ribosomal protein L9 | 148 |
| PF00281 | Ribosomal protein L5 | 56 |
| TIGR00981 | rpsL_bact: ribosomal protein S12 | 124 |
| TIGR00090 | iojap_ybeB: iojap-like ribosome-associated protein | 99 |
| TIGR00810 | secG: preprotein translocase, SecG subunit | 73 |
| TIGR00092 | TIGR00092: GTP-binding protein YchF | 368 |
| TIGR00095 | TIGR00095: RNA methyltransferase, RsmD family | 194 |
| TIGR00250 | RNAse_H_YqgF: RNAse H domain protein, YqgF family | 130 |
| TIGR01145 | ATP_synt_delta: ATP synthase F1, delta subunit | 172 |
| TIGR01029 | rpsG_bact: ribosomal protein S7 | 154 |
| TIGR03725 | T6A_YeaZ: tRNA threonylcarbamoyl adenosine modification protein YeaZ | 212 |
| TIGR01021 | rpsE_bact: ribosomal protein S5 | 156 |
| TIGR01024 | rplS_bact: ribosomal protein L19 | 114 |
| TIGR00061 | L21: ribosomal protein L21 | 101 |
| TIGR00337 | PyrG: CTP synthase | 526 |
| TIGR00060 | L18_bact: ribosomal protein L18 | 114 |
| TIGR01394 | TypA_BipA: GTP-binding protein TypA/BipA | 594 |
| PF10458 | Valyl tRNA synthetase tRNA binding arm | 66 |
| TIGR01393 | lepA: GTP-binding protein LepA | 595 |
| TIGR00436 | era: GTP-binding protein Era | 270 |
| TIGR00631 | uvrb: excinuclease ABC subunit B | 658 |
| TIGR00062 | L27: ribosomal protein L27 | 83 |
| TIGR00634 | recN: DNA repair protein RecN | 563 |
| TIGR00635 | ruvB: Holliday junction DNA helicase RuvB | 305 |
| TIGR00431 | TruB: tRNA pseudouridine(55) synthase | 210 |
| TIGR00472 | pheT_bact: phenylalanine--tRNA ligase, beta subunit | 798 |
| TIGR00496 | frr: ribosome recycling factor | 176 |
| TIGR03635 | S17_bact: 30S ribosomal protein S17 | 72 |
| PF01192 | RNA polymerase Rpb6 | 57 |
| PF02576 | Uncharacterised BCR, YhbC family COG0779 | 141 |
| TIGR01087 | murD: UDP-N-acetylmuramoylalanine--D-glutamate ligase | 441 |
| TIGR01169 | rplA_bact: ribosomal protein L1 | 227 |
| TIGR00487 | IF-2: translation initiation factor IF-2 | 587 |
| TIGR00922 | nusG: transcription termination/antitermination factor NusG | 172 |
| TIGR01164 | rplP_bact: ribosomal protein L16 | 126 |
| TIGR01009 | rpsC_bact: ribosomal protein S3 | 212 |
| TIGR00043 | TIGR00043: probable rRNA maturation factor YbeY | 111 |
| PF00466 | Ribosomal protein L10 | 100 |
| TIGR01128 | holA: DNA polymerase III, delta subunit | 314 |
| TIGR00166 | S6: ribosomal protein S6 | 95 |
| TIGR00416 | sms: DNA repair protein RadA | 454 |
| TIGR02386 | rpoC_TIGR: DNA-directed RNA polymerase, beta' subunit | 1147 |
| TIGR00344 | alaS: alanine--tRNA ligase | 847 |
| TIGR00615 | recR: recombination protein RecR | 196 |
| TIGR02273 | 16S_RimM: 16S rRNA processing protein RimM | 166 |
| TIGR00360 | ComEC_N-term: ComEC/Rec2-related protein | 171 |
| TIGR03654 | L6_bact: ribosomal protein L6 | 175 |
| TIGR01171 | rplB_bact: ribosomal protein L2 | 275 |
| TIGR00959 | ffh: signal recognition particle protein | 428 |
| TIGR01071 | rplO_bact: ribosomal protein L15 | 144 |
| TIGR00952 | S15_bact: ribosomal protein S15 | 86 |
| TIGR01079 | rplX_bact: ribosomal protein L24 | 104 |
| TIGR00116 | tsf: translation elongation factor Ts | 293 |
| TIGR00059 | L17: ribosomal protein L17 | 112 |
| PF00673 | ribosomal L5P family C-terminus | 95 |
| TIGR00593 | pola: DNA polymerase I | 890 |
| TIGR00595 | priA: primosomal protein N' | 509 |
| TIGR01632 | L11_bact: ribosomal protein L11 | 140 |
| TIGR02432 | lysidine_TilS_N: tRNA(Ile)-lysidine synthetase | 189 |
| TIGR00460 | fmt: methionyl-tRNA formyltransferase | 315 |
| TIGR00468 | pheS: phenylalanine--tRNA ligase, alpha subunit | 324 |
| TIGR01066 | rplM_bact: ribosomal protein L13 | 141 |
| TIGR01067 | rplN_bact: ribosomal protein L14 | 122 |
| PF00276 | Ribosomal protein L23 | 92 |
| TIGR00422 | valS: valine--tRNA ligase | 863 |
| TIGR00194 | uvrC: excinuclease ABC subunit C | 574 |
| TIGR02729 | Obg_CgtA: Obg family GTPase CgtA | 329 |
| TIGR00020 | prfB: peptide chain release factor 2 | 365 |
| PF13742 | OB-fold nucleic acid binding domain | 99 |
| TIGR03632 | bact_S11: 30S ribosomal protein S11 | 117 |
| TIGR00029 | S20: ribosomal protein S20 | 87 |
| TIGR03263 | guanyl_kin: guanylate kinase | 180 |
| TIGR01510 | coaD_prev_kdtB: pantetheine-phosphate adenylyltransferase | 155 |
| TIGR02013 | rpoB: DNA-directed RNA polymerase, beta subunit | 1238 |
| TIGR03631 | bact_S13: 30S ribosomal protein S13 | 113 |
| TIGR00091 | TIGR00091: tRNA (guanine-N(7)-)-methyltransferase | 194 |
| TIGR01050 | rpsS_bact: ribosomal protein S19 | 92 |
| TIGR00188 | rnpA: ribonuclease P protein component | 111 |
| TIGR00877 | purD: phosphoribosylamine--glycine ligase | 425 |
| TIGR01011 | rpsB_bact: ribosomal protein S2 | 225 |
| **Removed markers** | | |
| TIGR01951 | nusB: transcription antitermination factor NusB | 131 |
| TIGR00033 | aroC: chorismate synthase | 351 |
| TIGR00234 | tyrS: tyrosine--tRNA ligase | 406 |
| PF01416 | tRNA pseudouridine synthase | 105 |
| TIGR00447 | pth: peptidyl-tRNA hydrolase | 188 |
| TIGR00445 | mraY: phospho-N-acetylmuramoyl-pentapeptide-transferase | 321 |
| TIGR00539 | hemN_rel: putative oxygen-independent coproporphyrinogen III oxidase | 361 |
| TIGR00963 | secA: preprotein translocase, SecA subunit | 787 |
| TIGR00009 | L28: ribosomal protein L28 | 58 |
| TIGR00459 | aspS_bact: aspartate--tRNA ligase | 586 |
| TIGR00456 | argS: arginine--tRNA ligase | 569 |
| TIGR00008 | infA: translation initiation factor IF-1 | 69 |
| TIGR00083 | ribF: riboflavin biosynthesis protein RibF | 290 |
| TIGR00329 | gcp_kae1: metallohydrolase, glycoprotease/Kae1 family | 305 |
| TIGR01031 | rpmF_bact: ribosomal protein L32 | 56 |
| TIGR01034 | metK: methionine adenosyltransferase | 377 |
| TIGR00398 | metG: methionine--tRNA ligase | 530 |
| TIGR00420 | trmU: tRNA (5-methylaminomethyl-2-thiouridylate)-methyltransferase | 351 |
| TIGR00179 | murB: UDP-N-acetylenolpyruvoylglucosamine reductase | 290 |
| PF04127 | DNA / pantothenate metabolism flavoprotein | 185 |
| TIGR02027 | rpoA: DNA-directed RNA polymerase, alpha subunit | 298 |
| TIGR00382 | clpX: ATP-dependent Clp protease, ATP-binding subunit ClpX | 414 |
| TIGR00065 | ftsZ: cell division protein FtsZ | 353 |
| TIGR00064 | ftsY: signal recognition particle-docking protein FtsY | 279 |
| TIGR01391 | dnaG: DNA primase | 414 |
| TIGR00544 | lgt: prolipoprotein diacylglyceryl transferase | 280 |
| TIGR00739 | yajC: preprotein translocase, YajC subunit | 84 |
| TIGR01017 | rpsD_bact: ribosomal protein S4 | 200 |
| TIGR02191 | RNaseIII: ribonuclease III | 219 |
| PF07479 | NAD-dependent glycerol-3-phosphate dehydrogenase C-terminus | 149 |
| TIGR00174 | miaA: tRNA dimethylallyltransferase | 288 |
| TIGR02397 | dnaX_nterm: DNA polymerase III, subunit gamma and tau | 355 |
| TIGR01082 | murC: UDP-N-acetylmuramate--alanine ligase | 449 |
| TIGR01083 | nth: endonuclease III | 192 |
| TIGR01085 | murE: UDP-N-acetylmuramyl-tripeptide synthetase | 472 |
| TIGR00575 | dnlj: DNA ligase, NAD-dependent | 652 |
| TIGR00884 | guaA_Cterm: GMP synthase (glutamine-hydrolyzing), C-terminal domain | 310 |
| TIGR01162 | purE: phosphoribosylaminoimidazole carboxylase, catalytic subunit | 156 |
| PF01025 | GrpE | 166 |
| TIGR00233 | trpS: tryptophan--tRNA ligase | 328 |
| TIGR00186 | rRNA_methyl_3: RNA methyltransferase, TrmH family, group 3 | 240 |
| TIGR00165 | S18: ribosomal protein S18 | 70 |
| TIGR00414 | serS: serine--tRNA ligase | 418 |
| TIGR02075 | pyrH_bact: UMP kinase | 233 |
| TIGR00418 | thrS: threonine--tRNA ligase | 565 |
| TIGR00419 | tim: triose-phosphate isomerase | 228 |
| TIGR00362 | DnaA: chromosomal replication initiator protein DnaA | 437 |
| TIGR00888 | guaA_Nterm: GMP synthase (glutamine-hydrolyzing), N-terminal domain | 188 |
| TIGR00054 | TIGR00054: RIP metalloprotease RseP | 421 |
| TIGR00168 | infC: translation initiation factor IF-3 | 165 |
| TIGR00042 | TIGR00042: non-canonical purine NTP pyrophosphatase, RdgB/HAM1 family | 184 |
| TIGR00115 | tig: trigger factor | 410 |
| PF02601 | Exonuclease VII, large subunit | 319 |
| TIGR00663 | dnan: DNA polymerase III, beta subunit | 367 |
| PF00162 | Phosphoglycerate kinase | 384 |
| TIGR01063 | gyrA: DNA gyrase, A subunit | 800 |
| TIGR01060 | eno: phosphopyruvate hydratase | 425 |
| TIGR02727 | MTHFS_bact: 5-formyltetrahydrofolate cyclo-ligase | 182 |
| TIGR02350 | prok_dnaK: chaperone protein DnaK | 596 |
| TIGR00580 | mfd: transcription-repair coupling factor | 923 |
| TIGR03534 | RF_mod_PrmC: protein-(glutamine-N5) methyltransferase, release factor-specific | 253 |
| TIGR02012 | tigrfam_recA: protein RecA | 321 |
| TIGR00006 | TIGR00006: 16S rRNA (cytosine(1402)-N(4))-methyltransferase | 310 |
| TIGR00442 | hisS: histidine--tRNA ligase | 406 |
| TIGR01051 | topA_bact: DNA topoisomerase I | 632 |
| TIGR00967 | 3a0501s007: preprotein translocase, SecY subunit | 414 |
| PF00342 | Phosphoglucose isomerase | 486 |
| TIGR00643 | recG: ATP-dependent DNA helicase RecG | 629 |
| TIGR00392 | ileS: isoleucine--tRNA ligase | 861 |
