# Supplementary material for: Back from the dead; the curious tale of the predatory cyanobacterium Vampirovibrio chlorellavorus
Source: PeerJ. 2015 May 21;3:e968. doi: 10.7717/peerj.968 (PMC4451040; doi:10.7717/peerj.968)
Supplement: Table S2 — PHX and alien gene prediction was performed with PHX analysis, using ribosomal proteins, chaperones and transcriptional and translational proteins of V. chlorellavous as representatives of recognised highly expressed genes to identify other putatively highly expressed genes in the genome (Karlin & Mrázek, 2000). [file peerj-03-968-s009.docx]

**Highly expressed and alien gene**

**IMG number IMG annotation Eg number**

**2600256191** Protein-export membrane protein, SecD/SecF family 1.05

**Highly expressed genes**

**IMG number IMG annotation Eg number**

**2600254915** ATP synthase F1 subcomplex beta subunit 1.50

**2600254966** Superfamily II DNA and RNA helicases 1.10

**2600254993** Parvulin-like peptidyl-prolyl isomerase 1.17

**2600255027** DNA-binding protein, YbaB/EbfC family 1.20

**2600255064** Pyruvate:ferredoxin oxidoreductase and related 1.70

2-oxoacid:ferredoxin oxidoreductases, beta subunit

**2600255065** Pyruvate:ferredoxin oxidoreductase and related 1.32

2-oxoacid:ferredoxin oxidoreductases, alpha subunit

**2600255172** Thioredoxin-like proteins and domains 1.10

**2600255235** 5'-deoxy-5'-methylthioadenosine phosphorylase 1.45

**2600255249** Sugar transferases involved in lipopolysaccharide synthesis 1.05

**2600255283** P22 coat protein - gene protein 5 1.12

**2600255291** Vacuolar-type H(+)-translocating pyrophosphatase 1.69

**2600255297** Aspartyl-tRNA synthetase, bacterial type 1.35

**2600255302** Hypothetical protein 1.10

**2600255315** S-adenosylmethionine decarboxylase 1.28

**2600255327** Predicted P-loop-containing kinase 1.01

**2600255352** Chaperone protein DnaK 1.62

**2600255416** NADH dehydrogenase subunit A (EC 1.6.5.3) 1.11

**2600255450** Malate dehydrogenase (NAD) (EC 1.1.1.37) 1.13

**2600255470** Membrane protein insertase, YidC/Oxa1 family, 1.18

C-terminal domain

**2600255524** YtxH-like protein 1.02

**2600255533** Signal peptide peptidase SppA, 36K type 1.05

**2600255566** Outer membrane protein 1.16

**2600255576** Glyceraldehyde-3-phosphate dehydrogenase (NAD+) 1.32

(EC 1.2.1.12)

**2600255586** RNA polymerase sigma factor, sigma-70 family 1.41

**2600255592** Chaperonin GroL 1.86

**2600255626** Ribosomal protein L7/L12 1.43

**2600255627** DNA-directed RNA polymerase subunit beta (EC 2.7.7.6) 1.41

**2600255628** DNA-directed RNA polymerase gamma chain (EC 2.7.7.6) 1.49

**2600255637** Two component transcriptional regulator, LuxR family 1.10

**2600255664** Ribonucleotide reductase, beta subunit 1.14

**2600255681** ATPases with chaperone activity, ATP-binding subunit 1.21

**2600255723** Cbb3-type cytochrome oxidase, subunit 1 1.07

**2600255734** Hypothetical protein 1.08

**2600255835** Flagellar basal body L-ring protein 1.14

**2600255847** Superoxide dismutase 1.30

**2600255852** Ribosomal protein L9 1.41

**2600255862** Protein of unknown function (DUF561) 1.38

**2600255880** Septum site-determining protein MinD 1.07

**2600255892** Ribosomal protein S3, bacterial type 1.52

**2600255895** LSU ribosomal protein L2P 1.40

**2600255900**  Translation elongation factor 1A (EF-1A/EF-Tu) 1.41

**2600255911** Translation elongation factor EF-G 2.00

**2600255941** Rare lipoprotein A 1.24

**2600255966** Ribosomal protein L17 1.31

**2600255967** DNA-directed RNA polymerase, alpha subunit, bacterial 1.23

and chloroplast-type

**2600255975** LSU ribosomal protein L15P 1.34

**2600255977** Ribosomal protein S5, bacterial/organelle type 1.59

**2600256085** Polyribonucleotide nucleotidyltransferase 1.71

**2600256086** SSU ribosomal protein S15P 1.07

**2600256099** Translation elongation factor P 1.21

**2600256132** Phosphopentomutase 1.21

**2600256134** Uncharacterized protein conserved in bacteria 1.04

**2600256170** Uncharacterized conserved protein 1.05

**2600256171** 6-pyruvoyl-tetrahydropterin synthase 1.11

**2600256173** Bacterial regulatory proteins, gntR family 1.23

**2600256207** Enoyl-[acyl-carrier-protein] reductase [NADH] 1.30

(EC 1.3.1.9)

**2600256221** Hypothetical protein 1.16

**2600256238** Ribose-phosphate pyrophosphokinase 1.15

**2600256248** Hypothetical protein 1.28

**2600256265** Aconitase (EC 4.2.1.3) 1.11

**2600256266** Isocitrate dehydrogenase (NADP) (EC 1.1.1.42) 1.34

**2600256282** Peroxiredoxin 1.53

**2600256283**  Ribosomal protein L1, bacterial/chloroplast 1.47

**2600256284** LSU ribosomal protein L11P 1.19

**2600256314** S1 RNA binding domain 1.67

**2600256336** S1 RNA binding domain 1.38

**2600256348** Adenosylhomocysteinase (EC 3.3.1.1) 1.38

**2600256354** Carbon storage regulator, CsrA 1.18

**2600256361** NusA antitermination factor 1.15

**2600256362** Translation initiation factor IF-2 1.24

**2600256385** Bacterial SH3 domain 1.28

**2600256386** Hypothetical protein 1.84

**2600256388** Thioredoxin 1.36

**2600256401** Hypothetical protein 1.08

**2600256411** FKBP-type peptidyl-prolyl cis-trans isomerases 2 1.14

**2600256417** Inosine-5'-monophosphate dehydrogenase 1.06

(EC 1.1.1.205)

**2600256436** Glycosyl hydrolases family 8 1.10

**2600256437** Threonyl-tRNA synthetase (EC 6.1.1.3) 1.02

**2600256466** 6-phosphofructokinase 1.10

**2600256529** LL-diaminopimelate aminotransferase apoenzyme 1.27

(EC 2.6.1.83)

**2600256549** Aspartyl/glutamyl-tRNA(Asn/Gln) amidotransferase 1.12

subunit C (EC 6.3.5.-)

**2600256550** CTP synthase (EC 6.3.4.2) 1.10

**2600256560** Fe2+/Zn2+ uptake regulation proteins 1.11

**2600256561** Rubrerythrin 1.09

**2600256583** Two component transcriptional regulator, LuxR family 1.12

**2600256588** Nucleoside diphosphate kinase (EC 2.7.4.6) 1.41

**2600256597** Cyanobacterial porin (TC 1.B.23) 1.67

**2600256623** 2-oxoacid:acceptor oxidoreductase, alpha subunit 1.79

**2600256624** 2-oxoacid:acceptor oxidoreductase, beta subunit, 1.41

pyruvate/2-ketoisovalerate family

**2600256651** Flagellar hook-basal body protein 1.17

**2600256660** Biopolymer transport proteins 1.08

**2600256663** Hypothetical protein 1.09

**2600256675** 3-deoxy-D-arabinoheptulosonate-7-phosphate 1.03

synthase (EC 2.5.1.54)

**2600256710** GTP-binding protein TypA/BipA 1.46

**2600256731** Hypothetical protein 1.13

**2600256733** Adenine phosphoribosyltransferase (EC 2.4.2.7) 1.18

**2600256757** ATP synthase F1 subcomplex alpha subunit 1.31

**2600256779** Hemolysin activation/secretion protein 1.23

**2600256821** Phosphate transport regulator 1.05

**2600256833**  Hypothetical protein 1.06

**2600256897** YlqD protein 1.18

**2600256918** Exodeoxyribonuclease VII small subunit (EC 3.1.11.6) 1.06

**2600256944** S-layer homology domain 1.07

**2600256966** RNA polymerase, sigma 28 subunit, SigD/FliA/WhiG 1.38

**2600256998**  Glutaredoxin-related protein 1.14

**2600257077** Cell division protein FtsZ 1.28

**2600257160** Alpha-glucan phosphorylases 1.38

**2600257195** Peroxiredoxin, OsmC subfamily 1.43

**2600257223** Type III secretion system ATPase, FliI/YscN 1.08

**2600257249** Hypothetical protein 1.13

**2600257292** Protein of unknown function (DUF1292) 1.33

**2600257293** Succinyl-CoA synthetase (ADP-forming) beta subun 1.28

(EC 6.2.1.5)

**2600257294** Succinyl-CoA synthetase, alpha subunit 1.27

**2600257316** Ribosomal protein S2, bacterial type 1.79

**2600257317** Translation elongation factor Ts (EF-Ts) 1.15

**2600257354** Hypothetical protein 1.12

**2600257355** Hypothetical protein 1.10

**2600257409** ATP-dependent Clp protease ATP-binding subunit 1.00

ClpX (EC 3.4.21.92)

**2600257410** ATP-dependent Clp protease proteolytic subunit 1.10

ClpP (EC 3.4.21.92)

**2600257411**  Trigger factor 1.48

**2600257427** Succinate dehydrogenase/fumarate reductase, flavoprotein 1.30

subunit

**2600257445** Flagellar basal body rod protein 1.07

**2600257449** Flagellar basal-body rod protein FlgC 1.06

**Alien genes**

**IMG number IMG annotation Eg number**

**2600256772** Hypothetical protein 0.99

**2600255126** Hypothetical protein 0.98

**2600256735** Hypothetical protein 0.92

**2600255443** Hypothetical protein 0.88

**2600257671** Periplasmic protein involved in polysaccharide export 0.88

**2600257031** Hypothetical protein 0.86

**2600257567** Imidazoleglycerol-phosphate synthase 0.86

**2600256774** Beta-lactamase class C and other penicillin binding 0.85

proteins

**2600257020** Hypothetical protein 0.85

**2600257499** Hypothetical protein 0.85

**2600257685** Hypothetical protein 0.85

**2600255194** Hypothetical protein 0.84

**2600255922** FOG: CheY-like receiver 0.84

**2600256485** prepilin-type N-terminal cleavage/methylation domain 0.84

**2600257568** Imidazole glycerol phosphate synthase, glutamine 0.84

amidotransferase subunit

**2600256419** Hypothetical protein 0.83

**2600256973** Hypothetical protein 0.82

**2600257103** Outer membrane protein 0.82

**2600257113** Hypothetical protein 0.82

**2600257585** Transposase and inactivated derivatives 0.82

**2600257699** Conjugal transfer protein TrbH 0.82

**2600257732** AAA ATPase domain/AAA domain 0.82

**2600255868** Hypothetical protein 0.81

**2600257187** Twin arginine targeting (Tat) protein translocase TatC 0.81

**2600257497** Diadenosine tetraphosphate (Ap4A) hydrolase and other 0.81

HIT family hydrolases

**2600257527** Nucleotidyl transferase of unknown function (DUF1814) 0.81

**2600257529** Nucleotidyl transferase of unknown function (DUF1814) 0.81

**2600257563** Methyltransferase domain 0.81

**2600255144** Hypothetical protein 0.80

**2600255224** Hypothetical protein 0.80

**2600255777** TIR domain 0.80

**2600257632** Uncharacterized protein conserved in bacteria 0.80

**2600257676** Glycosyl transferase family 2 0.80

**2600254951** Hypothetical protein 0.79

**2600255590** Hypothetical protein 0.79

**2600255756** Hypothetical protein 0.79

**2600255862** Protein of unknown function (DUF561) 0.79

**2600256409** Domain of unknown function (DUF4145) 0.79

**2600256512** Alginate lyase 0.79

**2600257109** RHS repeat-associated core domain 0.79

**2600257362** Hypothetical protein 0.79

**2600257480** Plasmid encoded RepA protein 0.79

**2600257489** TaqI-like C-terminal specificity domain/ 0.79

Methyltransferase domain

**2600257491** Hypothetical protein 0.79

**2600257570** Hypothetical protein 0.79

**2600257594** Plasmid encoded RepA protein 0.79

**2600257630** Predicted ATP-dependent endonuclease of the OLD family 0.79

**2600257665** Hypothetical protein 0.79

**2600257691** Trypsin-like peptidase domain/PDZ domain 0.79

**2600255719** Hypothetical protein 0.78

**2600257105** RND family efflux transporter, MFP subunit 0.78

**2600257230** Dehydrogenases (flavoproteins) 0.78

**2600257492** Predicted ATPase (AAA+ superfamily) 0.78

**2600257669** Teichoic acid biosynthesis proteins 0.78

**2600255978** Predicted phosphohydrolases 0.77

**2600256525** Hypothetical protein 0.77

**2600256642** Hypothetical protein 0.77

**2600257225** Protein of unknown function (DUF2971) 0.77

**2600257562** Predicted pyridoxal phosphate-dependent enzyme 0.77

apparently involved in regulation of cell wall biogenesis

**2600257599** Domain of unknown function (DUF389) 0.77

**2600257674** Hypothetical protein 0.77

**2600257711** DnaA N-terminal domain 0.77

**2600256983** Peptidase M15 0.76

**2600257091** Hypothetical protein 0.76

**2600257561** Nucleoside-diphosphate-sugar epimerases 0.76

**2600257610** Uncharacterized conserved protein (COG2071) 0.76

**2600257667** Nucleoside-diphosphate-sugar pyrophosphorylase 0.76

involved in lipopolysaccharide biosynthesis/translation

initiation factor 2B

**2600257680** Endoglucanase 0.76

**2600255374** Hypothetical protein 0.75

**2600255506** Predicted phosphohydrolases 0.75

**2600257104** The (Largely Gram-negative Bacterial) 0.75

Hydrophobe/Amphiphile Efflux-1 (HAE1) Family

**2600257228** Predicted naringenin-chalcone synthase 0.75

**2600257508** Hypothetical protein 0.75

**2600257517** Hypothetical protein 0.75

**2600257663** Hypothetical protein 0.75

**2600257672** Uncharacterized protein involved in exopolysaccharide 0.75

biosynthesis

**2600257678** Glycosyltransferase 0.75

**2600255258** Dihydrofolate reductase 0.74

**2600255507** Type II secretion system (T2SS), protein F 0.74

**2600256033** Sugar transferases involved in lipopolysaccharide synthesis 0.74

**2600256720** Hypothetical protein 0.74

**2600257493** Adenine-specific DNA methylase containing a Zn-ribbon 0.74

**2600257537** RND family efflux transporter, MFP subunit 0.74

**2600257646** P-type conjugative transfer protein TrbL 0.74

**2600255269** Hypothetical protein 0.73

**2600257541** HipA-like C-terminal domain/HipA N-terminal domain/ 0.73

HipA-like N-terminal domain

**2600257572** ABC-type multidrug transport system, ATPase and 0.73

permease components

**2600257666** UDP-galactose 4-epimerase (EC 5.1.3.2) 0.73

**2600257677** Glycosyltransferase 0.73

**2600254929** Ankyrin repeats (3 copies) 0.72

**2600255090** Restriction endonuclease S subunits 0.72

**2600255508** Flp pilus assembly protein TadB 0.72

**2600256159** Glycosyltransferase 0.72

**2600257232** Ankyrin repeats (3 copies)/Ankyrin repeats 0.72

**2600255059** Hypothetical protein 0.71

**2600256873** Hypothetical protein 0.71

**2600257088** UvrD-like helicase C-terminal domain/UvrD/REP 0.71

helicase N-terminal domain

**2600257494** Hypothetical protein 0.71

**2600257607** DoxX-like family 0.71

**2600257673** Lipid A core - O-antigen ligase and related enzymes 0.71

**2600257675** Glycosyltransferases involved in cell wall biogenesis 0.71

**2600257686** Predicted ATPase (AAA+ superfamily) 0.71

**2600255060** Hypothetical protein 0.70

**2600255674** Translation factor SUA5 0.70

**2600257601** Trehalose-6-phosphate synthase 0.70

**2600255536** Hypothetical protein 0.68

**2600256155** Hypothetical protein 0.67

**2600256156** Glycosyltransferase 0.69

**2600257209** Ammonium transporter (TC 1.A.11) 0.69

**2600257275** Hypothetical protein 0.69

**2600257516** Type IV secretory pathway, VirB10 components 0.68

**2600256157** Coenzyme F390 synthetase 0.68

**2600256158** Coenzyme F390 synthetase 0.68

**2600256160** Membrane protein involved in the export of O-antigen 0.68

and teichoic acid

**2600256524** Hypothetical protein 0.68

**2600257575** Predicted dehydrogenase 0.68

**2600257231** Predicted membrane protein 0.67

**2600257569** Hypothetical protein 0.67

**2600257369** Hypothetical protein 0.66

**2600257564** Asparagine synthase 0.66

**2600257017** Dolichyl-phosphate-mannose-protein mannosyltransferase 0.64
