# Supplementary material for: Back from the dead; the curious tale of the predatory cyanobacterium Vampirovibrio chlorellavorus
Source: PeerJ. 2015 May 21;3:e968. doi: 10.7717/peerj.968 (PMC4451040; doi:10.7717/peerj.968)
Supplement: Table S3 — Putative gene numbers are assigned using IMG/ER (Markowitz et al., 2009). [file peerj-03-968-s010.docx]

**ORF number Gene Function**

***Flagella***

2600255340 *fliH* Flagellar assembly protein

2600255341 *flhA* Flagellar biosynthesis pathway

2600255378 *flgC* Flagella basal body rod protein

2600255418 *motB* Flagellar motor protein

2600255419 *motA* Flagellar motor component

2600255528 *flgE* flagellar hook-basal body protein

2600255530 *fliJ* flagellar export protein

2600255622 *fliS* flagellar biosynthetic protein

2600255623 *fliT* Flagellar protein

2600255743 *fliP* flagellar biosynthetic protein

2600255744 *fliQ* flagellar biosynthetic protein

2600255745 *fliR* flagellar biosynthetic protein

2600255746 *flhB* flagellar biosynthetic protein

2600255747 *flhA* flagellar biosynthesis protein

2600255829 *flgL* flagellar hook-associated protein 3

2600255830 *flgK* flagellar hook-associated protein

2600255831 *flgN* protein

2600255833 *flgJ* Rod binding protein

2600255834 *flgI* Flagellar basal-body P-ring protein

2600255835 *flgH* Flagellar basal body L-ring protein

2600255836 *flgA* flagella basal body P-ring formation protein

2600255837 *flgG* flagellar basal-body rod protein

2600255838 *flgG* flagellar hook-basal body protein

2600256111 *flgB* flagellar basal-body rod protein

2600256112 *flgC* flagellar basal-body rod protein

2600256113 *fliE* flagellar hook-basal body complex protein

2600256114 *fliF* flagellar basal-body M-ring protein/flagellar hook-basal body

protein

2600256115 *fliG* flagellar motor switch protein

2600256116 *fliH* Flagellar biosynthesis/type III secretory pathway protein

2600256117 *fliI* type III secretion system ATPase (EC 3.6.3.15)

2600256210 *fliN* flagellar motor switch protein

2600256211 *fliM* flagellar motor switch protein

2600256212 *fliL* Flagellar basal body-associated protein

2600256213 *motB* Flagellar motor protein

2600256214 *motA* Flagellar motor component

2600256257 *fliD* Flagellar capping protein

2600256427 *flhB* Flagellar biosynthesis pathway

2600256627 *fliR* Flagellar biosynthesis pathway

2600256628 *fliQ* Flagellar biosynthesis pathway

2600256629 *fliP* flagellar biosynthetic protein

2600256630 *fliO* Flagellar biosynthesis protein

2600256631 *spoA* Flagellar motor switch/type III secretory pathway protein

2600256648 *fliJ* Flagellar protein

2600256649 *fliK* Flagellar hook-length control protein

2600256650 *flgD* Flagellar hook capping protein - N-terminal region

2600256651 *flgE* flagellar hook-basal body protein

2600257074 *fliO* Flagellar biosynthesis protein

2600257445 *flgF* Flagellar basal body rod protein

2600257446 *flgG* flagellar hook-basal body protein

2600257447 *flgB* Flagellar basal body protein

2600257449 *flgC* flagellar basal-body rod protein

2600257451 *fliE* flagellar hook-basal body complex protein

2600257452 *fliF* Flagellar biosynthesis/type III secretory pathway lipoprotein

***Pili***

2600255034 *pulG* prepilin-type N-terminal cleavage/methylation domain

2600255035 *pulG* prepilin-type N-terminal cleavage/methylation domain

2600255080 *pulG* prepilin-type N-terminal cleavage/methylation domain

2600255146 *pulG* prepilin-type N-terminal cleavage/methylation domain

2600255147 *pulG* prepilin-type N-terminal cleavage/methylation domain

2600255148 *pulG* prepilin-type N-terminal cleavage/methylation domain

2600255219 *pulG* prepilin-type N-terminal cleavage/methylation domain

2600255220 *pulG* prepilin-type N-terminal cleavage/methylation domain

2600255349 *pulF* Type II secretory pathway, component PulF

2600255350 *pulE/pilB* Type II secretory pathway, ATPase PulE/Tfp pilus assembly pathway, ATPase PilB

2600255395 *tadD* Flp pilus assembly protein TadD, contains TPR repeats

2600255417 *tadD* Flp pilus assembly protein TadD, contains TPR repeats

2600255465 *pulG* prepilin-type N-terminal cleavage/methylation domain

2600255474 *pulG* prepilin-type N-terminal cleavage/methylation domain

2600255501 *tadD* Flp pilus assembly protein TadD, contains TPR repeats

2600255508 *tadB* Flp pilus assembly protein TadB

2600255509 *cpaF* Flp pilus assembly protein, ATPase CpaF

2600255510 *cpaE* Flp pilus assembly protein, ATPase CpaE

2600255511 *cpaC* Flp pilus assembly protein, secretin CpaC

2600255512 *cpaB* Flp pilus assembly protein CpaB

2600255572 *pulG* prepilin-type N-terminal cleavage/methylation domain

2600255587 *pulF/pilC* Type II secretory pathway, component PulF

2600255588 *pulE/pilB* Type II secretory pathway, ATPase PulE/Tfp pilus assembly pathway, ATPase PilB

2600255589 *pulD* Type II secretory pathway, component PulD

2600255642 *pilT* pilus retraction protein PilT

2600255666 - Flp pilus assembly protein, pilin Flp

2600255863 *pilF* Tfp pilus assembly protein PilF

2600255931 *pulG* prepilin-type N-terminal cleavage/methylation domain

2600255932 *pulG* prepilin-type N-terminal cleavage/methylation domain

2600255933 *pulG* prepilin-type N-terminal cleavage/methylation domain

2600255934 *pulG* prepilin-type N-terminal cleavage/methylation domain

2600256022 *pilF* Tfp pilus assembly protein PilF

2600256198 *pulG* prepilin-type N-terminal cleavage/methylation domain

2600256230 *pilV* Tfp pilus assembly protein PilV

2600256231 *pulG* prepilin-type N-terminal cleavage/methylation domain

2600256293 *pulG* prepilin-type N-terminal cleavage/methylation domain

2600256298 *tadE* TadE-like protein

2600256299 *cpaB* Flp pilus assembly protein CpaB

2600256300 - BON domain/Bacterial type II and III secretion system protein/Pilus formation protein N terminal region

2600256301 *cpaE* Flp pilus assembly protein, ATPase CpaE

2600256302 *cpaF* Flp pilus assembly protein, ATPase CpaF

2600256303 *tadB* Flp pilus assembly protein TadB

2600256304 *tadC* Flp pilus assembly protein TadC

2600256317 *pulO* Type II secretory pathway, prepilin signal peptidase PulO and related peptidases

2600256344 *pulG* prepilin-type N-terminal cleavage/methylation domain

2600256395 *pulG* prepilin-type N-terminal cleavage/methylation domain

2600256424 *pulG* prepilin-type N-terminal cleavage/methylation domain

2600256425 *pulG* prepilin-type N-terminal cleavage/methylation domain

2600256484 *pulG* prepilin-type N-terminal cleavage/methylation domain

2600256485 *pulG* prepilin-type N-terminal cleavage/methylation domain

2600256491 *pilT* pilus retraction protein PilT

2600256635 *tadD* Flp pilus assembly protein TadD, contains TPR repeats

2600256665 *pulG* prepilin-type N-terminal cleavage/methylation domain

2600256723 *pulG* prepilin-type N-terminal cleavage/methylation domain

2600256775 *pulG* prepilin-type N-terminal cleavage/methylation domain

2600256776 *pulG* prepilin-type N-terminal cleavage/methylation domain

2600256849 *pilF* Tfp pilus assembly protein PilF

2600256953 *pilN* Fimbrial assembly protein (PilN)

2600256956 *pilF* Tfp pilus assembly protein PilF

2600257134 *pulG* prepilin-type N-terminal cleavage/methylation domain

2600257156 *pulG* prepilin-type N-terminal cleavage/methylation domain

2600257173 *pulG* prepilin-type N-terminal cleavage/methylation domain

2600257271 *pulG* prepilin-type N-terminal cleavage/methylation domain

2600257291 *pulG* prepilin-type N-terminal cleavage/methylation domain

2600257309 *tadE/G* Putative Flp pilus-assembly TadE/G-like

2600257323 - Flp pilus assembly protein, pilin Flp

2600257324 - Flp pilus assembly protein, pilin Flp

2600257352 *pulG* prepilin-type N-terminal cleavage/methylation domain

2600257353 *pulG* prepilin-type N-terminal cleavage/methylation domain

2600257403 - Flp pilus assembly protein, pilin Flp
