# Supplementary material for: Back from the dead; the curious tale of the predatory cyanobacterium Vampirovibrio chlorellavorus
Source: PeerJ. 2015 May 21;3:e968. doi: 10.7717/peerj.968 (PMC4451040; doi:10.7717/peerj.968)
Supplement: Table S4 — Putative genes were annotated with the dbCAN web server (Yin et al., 2012) to identify glycoside hydrolases and checked against the IMG annotations and BLAST results. [file peerj-03-968-s011.docx]

**Known activity Pfam families and domains Number**

***Glycoside Hydrolases***

**Cellulases**

GH5 Endoglucanase PF00150 1

GH6 Endoglucanase PF01341 1

GH8 Endoglucanase PF01270 2

GH9 Endoglucanase PF02927/PF00759 1

**Murein degradation (T4SS)**

GH23 Transglycosylase PF01464 7

**Oligosaccharide-degrading enzymes**

GH3 Beta-galactosidase PF00933 1

GH35 Beta-galactosidase PF01301 1

GH38 Alpha-mannosidase PF01074/PF09261 1

**Polysaccharide-degrading enzymes**

GH13 Mainly α-amylase PF00128/PF02638/PF02806/PF02922/PF11941 7

GH77 4-α-glucanotransferase PF02446 1

**Other**

GH4 Glycerol-3-phosphate dehydrogenase PF01210/PF07479 1

GH15 Glucoamylase? PF00723 1

GH19 Chitinase PF01471 1

GH57 α-amylase, 4-α-glucanotransferase PF03065/PF09094 2

GH109 Oxidoreductase, 3-hydroxyisobutyrate PF01408/PF02894/PF03435/PF03446/PF14833 6

dehydrogenase, saccharopine dehydrogenase

***Glycosyltransferases***

GT1 - PF13528 1

GT2 - PF00534/PF00535/PF07238/PF13579/PF13641 18

GT4 Kdotransferase PF04413/PF00534/PF00535/PF13439/PF13579/

PF13477 17

GT5 - PF08323/PF00534 3

GT9 Heptosyltransferase PF01075 5

GT19 Lipid-A-disaccharide synthetase/ PF02684/PF02350 2

UDP-N-acetylglucosamine 2-epimerase

GT20 - PF00982 1

GT26 Glycosyl transferase WecB/TagA/CpsF PF02706/PF03808 5

GT27 - PF13641 1

GT28 Monogalactosyldiacylglycerol synthase PF03033/PF06925/PF13528 3

GT30 - PF00534/PF04413 1

GT35 Phosphorylase PF00343 2

GT39 Dolichyl-phosphate-mannose-protein PF13231 1

Mannosyltransferase

GT51 Transglycosylase/Transpeptidase PF00905/PF00912 1

GT83 Dolichyl-phosphate-mannose-protein PF13231 6

Mannosyltransferase

**Auxillary Activities**

AA2 Catalase/peroxidase PF00141 3

AA6 Flavoprotein PF03358 1

AA7 FAD/FMN-containing dehydrogenase PF01565/PF02913 2

**Carbohydrate-Binding Modules**

CBM48 1,4-alpha-glucan branching enzyme PF00128/PF02922/PF11941 2

CBM50 LysM domain PF01476 1

CBM57 Melectin PF11721 2

**Carbohydrate Esterases**

CE4 Polysaccharide deacetylase PF01522 1

CE7 Esterase/lipase/hydrolase PF12695 3

CE9 N-acetylglucosamine-6-phosphate PF01979 2 deacetylase/cytosine deaminase

CE10 Esterase/lipase PF12697/PF12695 2

CE11 UDP-3-O-acyl-N-acetylglucosamine PF03331 1

deacetylase

**Polysaccharide Lyases**

PL7 Alginate lyase PF08787 1
