# Supplementary material for: Back from the dead; the curious tale of the predatory cyanobacterium Vampirovibrio chlorellavorus
Source: PeerJ. 2015 May 21;3:e968. doi: 10.7717/peerj.968 (PMC4451040; doi:10.7717/peerj.968)
Supplement: Table S5 — The MEROPS server (Rawlings et al., 2014) was used to identify putative peptidases in V. chlorellavorus using batch BLAST. [file peerj-03-968-s012.docx]

***Aspartic***

Cytoplasmic 2

Cytoplasmic membrane 1

Unknown 1

***Cysteine***

Cytoplasmic 9

Unknown 5

***Serine***

Cytoplasmic 11

Cytoplasmic membrane 5

Periplasmic 5

Extracellular 1

Unknown 15

***Metallo***

Cytoplasmic 21

Cytoplasmic membrane 7

Periplasmic 3

Outer membrane 2

Unknown 12

***Inhibitor***

Cytoplasmic 1

Outer membrane 1

***NB***

Cytoplasmic 2

***Unknown***

Cytoplasmic 1
