# Supplementary material for: Back from the dead; the curious tale of the predatory cyanobacterium Vampirovibrio chlorellavorus
Source: PeerJ. 2015 May 21;3:e968. doi: 10.7717/peerj.968 (PMC4451040; doi:10.7717/peerj.968)
Supplement: Figure S3 — Three MCPs are encoded in the V. chlorellavorus genome. MCP domains were predicted with InterProScan5 (Jones et al., 2014). Directional arrows represent the putative genes and orientation (positive or negative strand) and the rounded rectangles represent domains. [file peerj-03-968-s003.pdf]

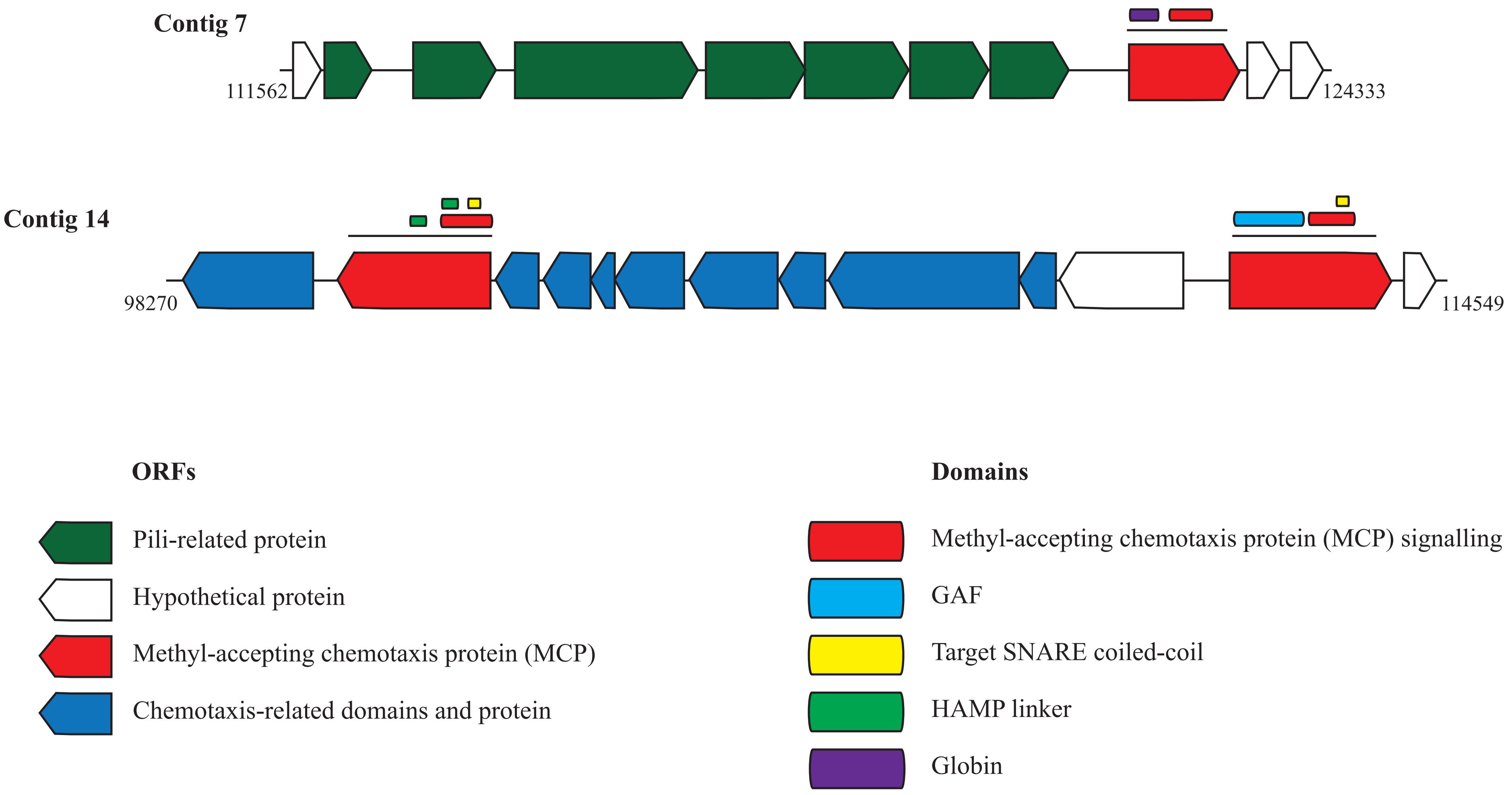
Contig 7
111562
124333
Contig 14
98270
114549
ORFs
Pili-related protein
Hypothetical protein
Methyl-accepting chemotaxis protein (MCP)
Chemotaxis-related domains and protein
Domains
Methyl-accepting chemotaxis protein (MCP) signalling
GAF
Target SNARE coiled-coil
HAMP linker
Globin
