# Supplementary material for: Back from the dead; the curious tale of the predatory cyanobacterium Vampirovibrio chlorellavorus
Source: PeerJ. 2015 May 21;3:e968. doi: 10.7717/peerj.968 (PMC4451040; doi:10.7717/peerj.968)
Supplement: Figure S7 — There is an increase in the COGs associated with cell motility (category N) and intracellular trafficking, secretion, and vesicular transport (category U) for V. chlorellavorus. STAMP (Parks et al., 2014a; Parks et al., 2014b) was used to explore the resulting COG profiles and create summary plots. [file peerj-03-968-s007.pdf]

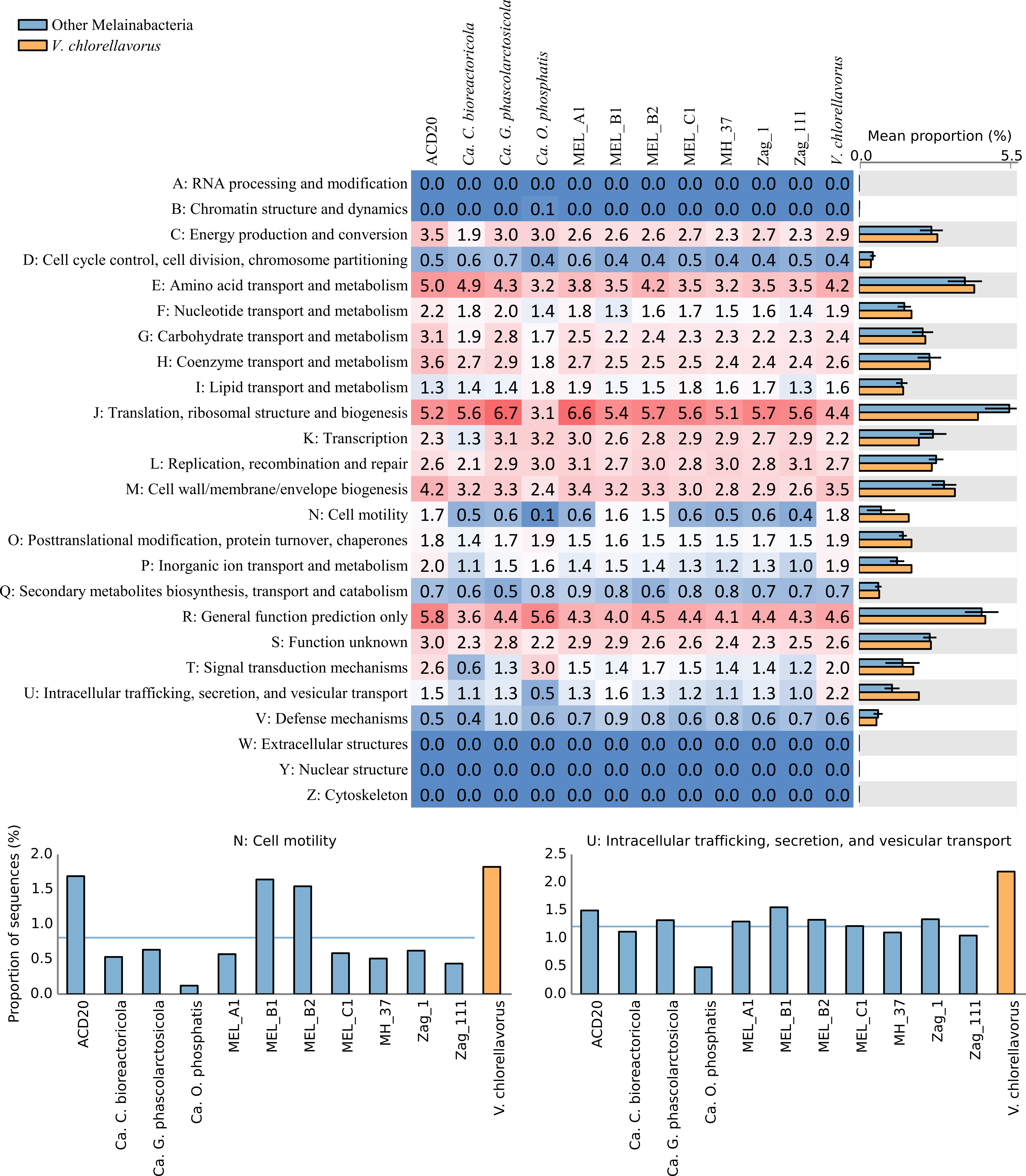
Other Melainabacteria
V. chlorellavorus
ACD20
Ca. C. bioreactoricola
Ca. G. phascolarctosicola
Ca. O. phosphatis
MEL_A1
MEL_B1
MEL_B2
MEL_C1
MH_37
Zag_1
Zag_111
V. chlorellavorus
Mean proportion (%)
0.0
5.5
A: RNA processing and modification 0.0 0.0 0.0 0.0 0.0 0.0 0.0 0.0 0.0 0.0 0.0 0.0
B: Chromatin structure and dynamics 0.0 0.0 0.0 0.1 0.0 0.0 0.0 0.0 0.0 0.0 0.0 0.0
C: Energy production and conversion 3.5 1.9 3.0 3.0 2.6 2.6 2.6 2.7 2.3 2.7 2.3 2.9
D: Cell cycle control, cell division, chromosome partitioning 0.5 0.6 0.7 0.4 0.6 0.4 0.4 0.5 0.4 0.4 0.5 0.4
E: Amino acid transport and metabolism 5.0 4.9 4.3 3.2 3.8 3.5 4.2 3.5 3.2 3.5 3.5 4.2
F: Nucleotide transport and metabolism 2.2 1.8 2.0 1.4 1.8 1.3 1.6 1.7 1.5 1.6 1.4 1.9
G: Carbohydrate transport and metabolism 3.1 1.9 2.8 1.7 2.5 2.2 2.4 2.3 2.3 2.2 2.3 2.4
H: Coenzyme transport and metabolism 3.6 2.7 2.9 1.8 2.7 2.5 2.5 2.5 2.4 2.4 2.4 2.6
I: Lipid transport and metabolism 1.3 1.4 1.4 1.8 1.9 1.5 1.5 1.8 1.6 1.7 1.3 1.6
J: Translation, ribosomal structure and biogenesis 5.2 5.6 6.7 3.1 6.6 5.4 5.7 5.6 5.1 5.7 5.6 4.4
K: Transcription 2.3 1.3 3.1 3.2 3.0 2.6 2.8 2.9 2.9 2.7 2.9 2.2
L: Replication, recombination and repair 2.6 2.1 2.9 3.0 3.1 2.7 3.0 2.8 3.0 2.8 3.1 2.7
M: Cell wall/membrane/envelope biogenesis 4.2 3.2 3.3 2.4 3.4 3.2 3.3 3.0 2.8 2.9 2.6 3.5
N: Cell motility 1.7 0.5 0.6 0.1 0.6 1.6 1.5 0.6 0.5 0.6 0.4 1.8
O: Posttranslational modification, protein turnover, chaperones 1.8 1.4 1.7 1.9 1.5 1.6 1.5 1.5 1.5 1.7 1.5 1.9
P: Inorganic ion transport and metabolism 2.0 1.1 1.5 1.6 1.4 1.5 1.4 1.3 1.2 1.3 1.0 1.9
Q: Secondary metabolites biosynthesis, transport and catabolism 0.7 0.6 0.5 0.8 0.9 0.8 0.6 0.8 0.8 0.7 0.7 0.7
R: General function prediction only 5.8 3.6 4.4 5.6 4.3 4.0 4.5 4.4 4.1 4.4 4.3 4.6
S: Function unknown 3.0 2.3 2.8 2.2 2.9 2.9 2.6 2.6 2.4 2.3 2.5 2.6
T: Signal transduction mechanisms 2.6 0.6 1.3 3.0 1.5 1.4 1.7 1.5 1.4 1.4 1.2 2.0
U: Intracellular trafficking, secretion, and vesicular transport 1.5 1.1 1.3 0.5 1.3 1.6 1.3 1.2 1.1 1.3 1.0 2.2
V: Defense mechanisms 0.5 0.4 1.0 0.6 0.7 0.9 0.8 0.6 0.8 0.6 0.7 0.6
W: Extracellular structures 0.0 0.0 0.0 0.0 0.0 0.0 0.0 0.0 0.0 0.0 0.0 0.0
Y: Nuclear structure 0.0 0.0 0.0 0.0 0.0 0.0 0.0 0.0 0.0 0.0 0.0 0.0
Z: Cytoskeleton 0.0 0.0 0.0 0.0 0.0 0.0 0.0 0.0 0.0 0.0 0.0 0.0
N: Cell motility
Proportion of sequences (%)
2.0
1.5
1.0
0.5
0.0
ACD20
Ca. C. bioreactoricola
Ca. G. phascolarctosicola
Ca. O. phosphatis
MEL_A1
MEL_B1
MEL_B2
MEL_C1
MH_37
Zag_1
Zag_111
V. chlorellavorus
U: Intracellular trafficking, secretion, and vesicular transport
2.5
2.0
1.5
1.0
0.5
0.0
ACD20
Ca. C. bioreactoricola
Ca. G. phascolarctosicola
Ca. O. phosphatis
MEL_A1
MEL_B1
MEL_B2
MEL_C1
MH_37
Zag_1
Zag_111
V. chlorellavorus
